# Construction of a High-Density Genetic Map from RNA-Seq Data for an Arabidopsis Bay-0 × Shahdara RIL Population

**DOI:** 10.3389/fgene.2017.00201

**Published:** 2017-12-05

**Authors:** Elise A. R. Serin, L. B. Snoek, Harm Nijveen, Leo A. J. Willems, Jose M. Jiménez-Gómez, Henk W. M. Hilhorst, Wilco Ligterink

**Affiliations:** ^1^Wageningen Seed Lab, Laboratory of Plant Physiology, Wageningen University, Wageningen, Netherlands; ^2^Laboratory of Nematology, Wageningen University, Wageningen, Netherlands; ^3^Theoretical Biology and Bioinformatics, Utrecht University, Utrecht, Netherlands; ^4^Laboratory of Bioinformatics, Wageningen University, Wageningen, Netherlands; ^5^Department of Plant Breeding and Genetics, Max Planck Institute for Plant Breeding Research, Cologne, Germany; ^6^Institut Jean-Pierre Bourgin, Institut National de la Recherche Agronomique, AgroParisTech, Centre National de la Recherche Scientifique, Université Paris-Saclay, Versailles Cedex, France

**Keywords:** Arabidopsis, genetic map, genotyping by sequencing, QTL mapping, RIL population, resolution, RNA-seq

## Abstract

High-density genetic maps are essential for high resolution mapping of quantitative traits. Here, we present a new genetic map for an Arabidopsis Bayreuth × Shahdara recombinant inbred line (RIL) population, built on RNA-seq data. RNA-seq analysis on 160 RILs of this population identified 30,049 single-nucleotide polymorphisms (SNPs) covering the whole genome. Based on a 100-kbp window SNP binning method, 1059 bin-markers were identified, physically anchored on the genome. The total length of the RNA-seq genetic map spans 471.70 centimorgans (cM) with an average marker distance of 0.45 cM and a maximum marker distance of 4.81 cM. This high resolution genotyping revealed new recombination breakpoints in the population. To highlight the advantages of such high-density map, we compared it to two publicly available genetic maps for the same population, comprising 69 PCR-based markers and 497 gene expression markers derived from microarray data, respectively. In this study, we show that SNP markers can effectively be derived from RNA-seq data. The new RNA-seq map closes many existing gaps in marker coverage, saturating the previously available genetic maps. Quantitative trait locus (QTL) analysis for published phenotypes using the available genetic maps showed increased QTL mapping resolution and reduced QTL confidence interval using the RNA-seq map. The new high-density map is a valuable resource that facilitates the identification of candidate genes and map-based cloning approaches.

## Introduction

Quantitative trait locus (QTL) analysis has successfully identified a large number of genetic loci that contribute to the regulation of quantitative phenotypes. The advent of -omics data has extended the range of usual mapping traits to molecular phenotypes offering new approaches for bridging the gap between genes and their function (Keurentjes et al., 2008). The idea that variation in gene expression can be treated as a quantitative trait gave rise to the concept of genetical genomics (Jansen and Nap, 2001). In combination with a genetic map, quantitative variation in gene expression measured in a segregating population enables the identification of expression QTLs (eQTLs). Many eQTL studies have contributed to our understanding of the genetic architecture of regulatory variation of intricate traits in Arabidopsis West (Keurentjes et al., 2007; West et al., 2007; Terpstra et al., 2010; Snoek et al., 2012; Lowry et al., 2013; Cubillos et al., 2014) (for review see Joosen et al., 2009), poplar (Drost et al., 2015), tomato (Ranjan et al., 2016), as well as in other organisms (Li et al., 2006, 2010; Rockman et al., 2010; Vinuela et al., 2010; Aylor et al., 2011; King et al., 2014; Snoek et al., 2017; Sterken et al., 2017).

In essence, the success of QTL mapping is determined by the mapping resolution which mainly depends on the size of the population (and thus the number of recombination events), the complexity of the phenotype, and the number of available markers. High-density genetic maps are thus instrumental for accurate mapping of QTLs. Traditional methods used to obtain molecular markers were mainly PCR based (SSR, AFLP, RFLP). New methods to derive molecular markers have recently emerged, together with the advancement of high-throughput technologies. Particularly, single-nucleotide polymorphisms (SNPs) represent a rich source of potential markers due to their abundance (Alonso-Blanco et al., 2016). Differences in gene expression measured with microarrays as a result of probe hybridization sensitivity to underlying sequence polymorphisms have been used to derive SNP-based markers (West et al., 2006; Zych et al., 2015, 2017). More recently, next-generation sequencing technologies for transcriptome analysis (RNA-seq) have provided unprecedented opportunities for quantitative genetics in plants (Jimenez-Gomez, 2011). Becoming a standard for gene expression profiling, RNA-seq has also proven to be an efficient and cost-effective method to identify genome-wide SNPs (Piskol et al., 2013; Markelz et al., 2017). In the context of genetical genomics, RNA-seq on a segregating population can simultaneously provide the molecular phenotype and the sequence information for molecular markers that subsequently provide genotyping information for the population.

Segregating bi-parental populations such as recombinant inbred line (RIL) populations are powerful tools for QTL analysis (Koornneef et al., 2004). These immortal populations capture frequent recombination events in a relatively small sized population, thereby conveniently reducing the costs for genotyping. In this study, we utilized an *Arabidopsis thaliana* Bayreuth (Bay 0) × Shahdara (Sha) population that has been used extensively for genetic (Loudet et al., 2002; Jimenez-Gomez et al., 2010) and eQTL studies (Keurentjes et al., 2007; West et al., 2007). The original genetic map for this population consists of 69 markers segregating in 420 F6 RILs (Loudet et al., 2002). Further genotyping efforts on a subset of these RILs have introduced markers derived from gene expression data with microarrays, saturating the original map (West et al., 2006; Salathia et al., 2007; Zych et al., 2015). Here, we present the construction of a high-resolution genetic map from RNA-seq data of 160 RILs. We validate and show the improvements of this new map by performing a QTL analysis with publicly available phenotypic data (Joosen et al., 2012).

## Materials and Methods

### Plant Growth and Sample Preparation

Seeds from the *A. thaliana* accessions Bay-0 and Sha and a Bay-0 × Sha RIL population consisting of 165 lines were used. This population was initially developed by Loudet et al. (2002). As part of a larger experiment aiming to investigate genotype × environment interactions, the parental lines and the RILs were grown under standard and controlled mild stress conditions. In the standard condition, plants were grown under long day (16 h light/8 h dark) at 70% RH and 22°C/18°C (day/night) under artificial light (150 μmol m^-2^ s^-1^). The plants were watered with a standard nutritive solution (see Supplementary Table S1 in He et al., 2014) three times a week by flooding cycles. The same conditions were used for the stress environments, except for the varying parameter as indicated hereafter: high temperature (25°C day/23°C night), high light (300 μmol m^-2^ s^-1^), and low phosphate (12.5 μM phosphate instead of 0.5 mM in the standard nutritive solution).

The RILs and the parental lines were first grown with three to four plants per environment in a single climate cell under the control conditions mentioned above. When most of the plants flowered, the main stems of all plants were removed to increase the numbers of side branches and thereby seed production, and to ensure that all seeds would complete their development under the specific conditions. Subsequently the plants were transferred to different climate cells to continue their growth under the specific stress conditions. At the time all plants in a given condition produced a sufficient amount of fully matured seeds; the seeds were bulk harvested from the three to four plants per line. After drying, a fraction of the freshly harvested seeds were stored at -80°C in sealed 2 ml tubes until RNA-seq library preparation.

### RNA Isolation and Sequencing

RNA was isolated from 4 to 5 mg of fresh harvested dry seeds that were stored at -80°C. Each of the parents was measured in triplicate per condition, i.e., 4 × 3 = 12 replicates per parent. RNA was extracted from the seeds of 160 RILs selected in conformity to the generalized genetical genomics (GGG) strategy (Li et al., 2008; Supplementary Table S1). RNA was isolated using the NucleoSpin RNA Plant Isolation Kit (Macherey-Nagel 740949) but adding Plant RNA isolation Aid (Life Technologies) according to the manufacturer’s protocol and instructions.

### RNA-Seq Reads Processing

Strand-specific RNA-seq libraries were prepared from each RNA sample using the TruSeq RNA kit from Illumina according to manufacturer’s instructions. Poly-A-selected mRNA was sequenced using the Illumina HiSeq2500 sequencer, producing strand-specific single-end reads of 100 nucleotides. Reads were trimmed using Trimmomatic (version 0.33, Bolger et al., 2014) to remove low quality nucleotides. Trimmed reads were subsequently mapped to the *A. thaliana* TAIR10 reference genome (Lamesch et al., 2012) using the HISAT2 software (version 2.0.1, Kim et al., 2015) with the “transcriptome mapping only” option. SNPs were called using the mpileup function of samtools (version 0.1.19, Li et al., 2009) and bcftools. The raw sequence data have been uploaded to the NCBI under the project identifier: PRJNA418075^1^.

### SNP Identification and RIL Genotyping

Variant call format (VCF) files were generated for each of the samples. Since not all SNPs are found in all genotypes, all vcf files were merged to generate a list with all variants present in at least one sample. From this unique list, information regarding the position in base pairs and the chromosome location of each SNP was retrieved and filtered for being consistent across the sequencing data of the parental lines. In order to get a more reliable genotypic score, canceling out any SNPs miscalls, and to reduce the overall number of markers, SNPs were grouped into bins. 1059 equal size artificial bins of 100 kbp were created along the whole genome. The scoring of the genotype was obtained based on the SNP information within each bin. For regions at the transition between two genotypic blocks, the bin score was rounded up and assigned to the closest genotypic score. The quality of the genotype scoring of the bins was assessed by correlation analysis.

### Nomenclature

The bins are ordered based on the genome sequence, thus the unit distance is not expressed in centimorgans (cM) but in bins of 100 kbp. Each bin is used as a marker and the midpoint position of the 100 kbp bin is used as the marker position. Markers were named RSM for RNA-seq markers, followed by the chromosome number of their location and their physical position in mega base pairs (Mbp). As an example RSM_1_0.05 corresponds to the marker at 0.05 Mbp on chromosome 1.

### Genetic Map Construction

The genetic distances in centimorgans of the 1059 markers for 160 RILs were estimated in order to describe and compare the new genetic map to previous maps. The genetic distances were estimated using the “est.map” function with “kosambi” distance from the R/qtl package (Broman et al., 2003; Arends et al., 2010). The correct order of the markers was verified by pairwise marker linkage analysis using the “est.rf” function. The recombination rate was determined based on the linear relation between the genetic and the physical positions of the marker. The segregation pattern was tested for all markers to identify markers that show significant distortion at the 5% level, after a Bonferroni correction for multiple testing. The statistical programming language R (version 3.3.2) (R Development Core Team, 2008) was used for all analyses. The genetic map and genotypic data are available in Supplementary Table S2.

### QTL Comparison

To test the effect of increased marker coverage on QTL mapping, we re-mapped 510 published phenotypic traits using the RNA-seq (1059 markers), the pheno2geno (497 markers) (Zych et al., 2015) and the original map (69 markers) (Loudet et al., 2002). In order to compare the mapping resolution, the genetic distances were re-estimated for each map using 145 RILs common to the three studies (Supplementary Table S1). The scanone function in R/qtl was used with the default settings for the QTL mapping. LOD score peaks were called by chromosome for each trait, resulting in a total of 2550 (510^∗^5) peak LOD scores. The LOD threshold for the genome-wide significance at the level of 5% was determined after 1000 permutations using each map. The LOD thresholds obtained were 2.36, 2.64, and 2.76 using the original, pheno2geno, and RNA-seq map, respectively. The increased LOD thresholds for the Pheno2geno and the RNA-seq map can be explained by the larger number of markers which will result in a larger multiple testing correction. We used a stringent LOD threshold of 3 to identify and compare significant QTLs for all maps. The LOD score comparison was performed in a similar way as described in Zych et al. (2015). To be more confident about the comparison, QTLs were considered to have a higher or lower LOD score if the difference between the compared LOD scores was larger or equal to 0.5. The mapping resolution of the RNA-seq map was investigated by comparing the confidence intervals (CIs) of QTLs for the RNA-seq and the original map. LOD-1 CIs were determined for all significant QTLs (LOD > 3) for both maps. The genomic positions of the lower and upper limit of each CI were estimated from the equation of the linear relation between genetic and physical position of the markers. Subsequently, the CI width was determined for each QTL in Mbp. The analyses and figures were generated using Microsoft Excel, R/qtl, and the R ggplot2 package.

The cross object containing all data for the 510 phenotypes in the 160 RILs for the QTL analysis is available in Supplementary Table S3. The QTL results for the comparison of the LOD scores and CIs are provided in Supplementary Tables S4 and S5. QTL profiles of the re-mapped 510 traits are available for interactive analysis in AraQTL^2^ (Nijveen et al., 2017).

## Results

### Genotyping the RIL Population Using a SNP Binning Approach

**FIGURE 1:**
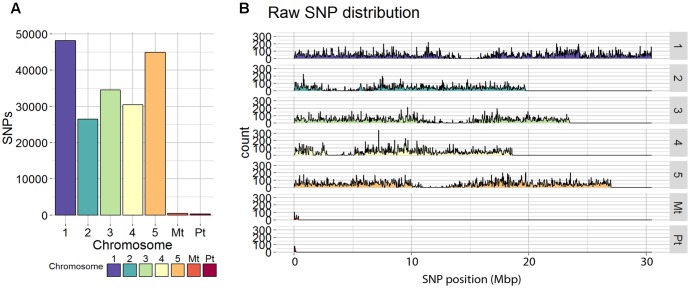
Raw SNP distribution from all genotyped RILs. **(A)** Total SNP count and **(B)** coverage counts of each SNP at each physical position on the chromosome in mega base pairs (Mbp) are displayed for each of the five chromosomes of *Arabidopsis thaliana* as well as the mitochondrial (Mt) and plastid (Pt) genomes.

Single-nucleotide polymorphisms calling resulted in 185,354 SNPs distributed over the five chromosomes, ranging from 26,514 SNPs for chromosome 2 to 48,151 SNPs for chromosome 1 (**Figure 1**). Regions with a few or no SNPs correspond to centromeric regions, known to have lower transcriptional density and expression activity (Schmid et al., 2005). Filtering and quality check of the SNPs (as described in the section “Materials and Methods”) resulted in a final number of 30,049 SNPs covering the whole genome.

The 100 kbp binning approach used, collapsed the 30,049 SNPs into 1059 bins distributed over the five chromosomes. Each bin contained on average ∼24 SNPs, with a minimum of 2 and a maximum of 130 SNPs per bin (Supplementary Figure S1). Overall, 96.7% of the bins could unambiguously be assigned to one of the parental genotypes.

**FIGURE 2:**
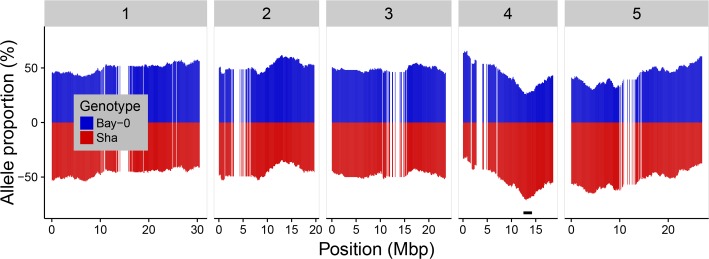
Allele distribution for the 1059 markers along the five chromosomes. Blue and red colors indicated the Bay-0 and the Sha allele percentages, respectively. The black horizontal bar indicates the region on chromosome 4 with 29 markers showing significant segregation distortion (*p*-value < 0.05 after Bonferroni correction).

Population-based SNPs segregated at the expected allele frequencies as global allelic equilibrium was observed with 49.3% Bay-0 alleles and 50.7% Sha alleles. Bias in the segregation ratio between the parental alleles was analyzed along the chromosomes (**Figure 2**). Statistically significant distortion of segregation was observed for 29 consecutive markers on chromosome 4, representing 2.78% of the total number of markers. These distorted markers correspond to the region comprised between the markers RSM_4_12.05 and RSM_4_14.85. The highest distortion was observed at the marker RSM_4_13.05 with 41 (25.6%) lines representing the Bay-0 allele versus 114 (114/160 = 71.25%) lines representing the Sha allele. This deviation from the allelic equilibrium at the chromosome 4 was also reported by Loudet et al. (2002).

### RNA-Seq Genotyping Identifies New Introgressions

**FIGURE 3:**
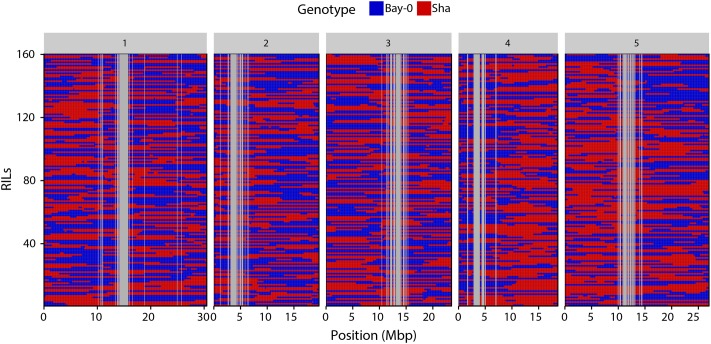
Haplotype representation of the 160 RILs. Each row corresponds to a RIL. Columns represent the 1059 genetic markers physically anchored on the five chromosomes. Blue boxes indicate Bay-0 genotype and red boxes indicate Sha genotypes.

Visually, the binning method resulted in the identification of clear genotype blocks (**Figure 3**). Breakpoints were identified as the point of transition between two genotype blocks. In total, 1455 crossovers were identified with an average of 291 crossovers per chromosome (**Table 1**).

**Table 1:** Characteristics of the 1059 marker genetic map using 160 RILs.

Chr	Markers	Total length (cM)	Average marker distance (cM)	Maximum gap (cM)	cross-overs	Recombination rate (kbp/cM)
chr 1	275	117.87	0.43	2.87	364	258.34
chr 2	171	76.29	0.45	3.23	236	257.58
chr 3	207	82.61	0.40	2.72	255	283.85
chr 4	163	92.12	0.57	4.81	281	201.36
chr 5	243	102.81	0.42	2.34	319	262.13
Total	Total 1059	Total 471.70	Average 0.45	Max 4.81	Total 1455	Average 252.65

To identify introgressions that were previously not detected, the 1059 new markers together with the 69 “old” markers were first ordered based on their physical positions. New introgressions were then identified in the RILs as double recombination events occurring within a region spanned by two “old” flanking markers and of a minimum size of 200 kbp (two bins) (Supplementary Figure S2). We could identify 80 unambiguous introgressions with sizes ranging between 200 kbp and 3 Mbp, increasing the number of recombination events detected within the RIL population.

### High-Density Genetic Map

Using each bin as a marker, the linkage map was calculated in order to validate the order of the markers and evaluate the accuracy of the new map. The characteristics of the new map are reported in **Table 1**. The total length of the genetic map was 471.70 cM. The average genetic distance between two adjacent markers of 0.45 cM represents a great increase in marker density as compared to the 6.1 cM of the 69 markers map for 420 RILs (Loudet et al., 2002). In the new map, the largest gap between two markers is 4.81 cM between the markers RSM_4_1.55 and RSM_4_1.85 on chromosome 4.

**FIGURE 4:**
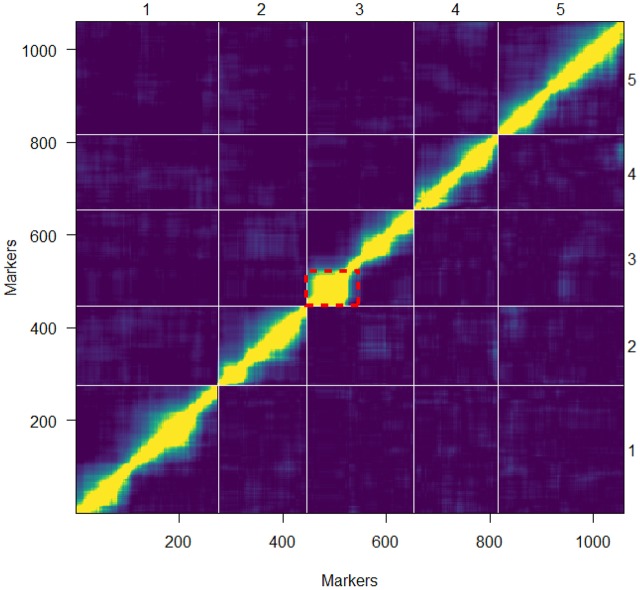
Pairwise marker linkage analysis. The estimated recombination fraction and LOD scores for all pairs of markers are shown in the upper-left and lower-right triangle, respectively. High correlation between markers indicates marker linkage (yellow) while the blue color shows low correlation values indicating unlinked markers. The grid delineates the five chromosomes. The red dotted frame indicates the region at the top of chromosome 3 with the probable occurrence of an inversion.

**FIGURE 5:**
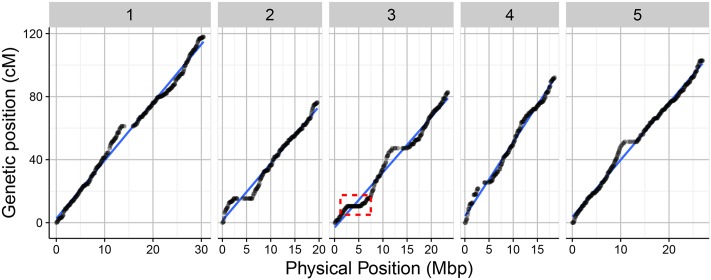
Relation between the genetic length in centimorgans (cM) and the physical length in Mbp for the 1059 markers along the five chromosome using 160 RILs of the Bay-0 × Sha RIL population. The red dotted frame indicates the region on the upper arm of chromosome 3 without recombination events.

Overall, the order of the markers on the genetic map conforms to the physical position of the marker and is also supported by the pairwise marker linkage analysis (**Figure 4**). The recombination rate was calculated as the relation between the physical and genetic distances. Low recombination was observed at the centromeric regions where the physical distance was greater relative to the genetic distance. On the upper arm of chromosome 3, no recombination events occurred between the markers RSM_3_2.65 and RSM_3_5.25. This was also observed in the 69-markers map as well as in a Sha × Col-0 RIL population^3^. A Sha-specific chromosomal inversion in this region was suggested (**Figures 4**, **5**). The global recombination rate is 252.65 kbp/cM, i.e., 4.01 cM per 1 Mbp (**Figure 5**). This rate is consistent with previously reported recombination rate of 246 kbp/cM (Loudet et al., 2002).

### QTL Mapping Comparison

The original genetic map for the analyzed Bay × Sha population developed by Loudet et al. (2002) comprises 69 PCR-based markers. Recently, Zych et al. (2015) saturated the original map with 497 markers derived from microarray expression data (pheno2geno map). To compare the published maps to the RNA-seq map, the genetic distances were re-estimated using 145 RILs common to the three studies (Supplementary Table S1).

**FIGURE 6:**
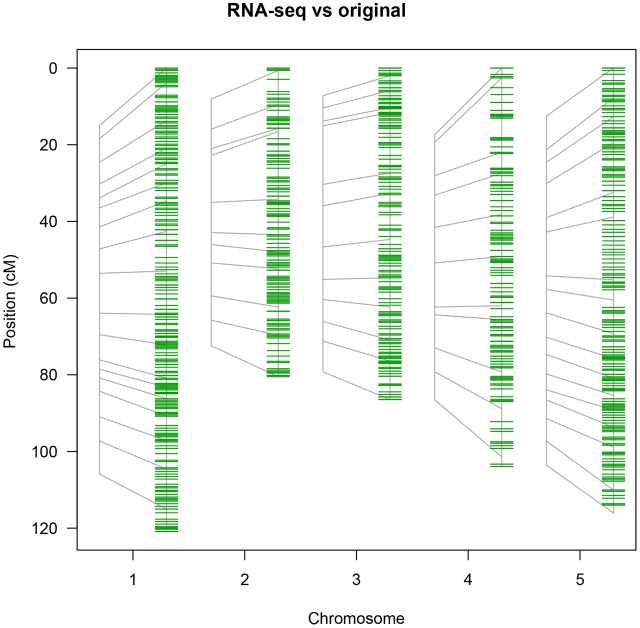
Saturation of the original map (69 PCR-based markers) with RNA-seq-derived markers. The position of the original markers is represented on the left of each chromosome in gray and linked to their position in the saturated map (green markers).

The RNA-seq map reduces the average distance between markers from 7.5 cM for the 69 marker map and 1 cM for the pheno2geno map to 0.6 cM (**Table 2**), closing many existing gaps in marker coverage (**Figure 6**). In addition, the RNA-seq map captures 1297 crossovers as compared to 1137 in the original map. The number of crossovers observed with the pheno2geno map (1366 cross-overs) is likely inflated due to the imputation of the genotypic data to 100% (% genotyped in **Table 2**).

**Table 2:** Summary of genetic maps for the Bay-0 × Sha RIL population based on145 RILs.

Genetic map parameters	Original	Pheno2Geno	RNA-seq
Number of markers	69	497	1059
Total length (cM)	480.1	499.1	464.4
Average marker distance (cM)	7.5	1	0.6
Maximum gap	22.9	11.6	4.9
Number of crossovers	1137	1366	1297
% genotyped	96.2	100	96.6
Global allele equilibrium	Bay 50.6%	Bay 49.7%	Bay 49.8%
	Sha 49.4%	Sha 50.3%	Sha 50.2%
Reference	Loudet et al., 2002	Zych et al., 2015	This study

**FIGURE 7:**
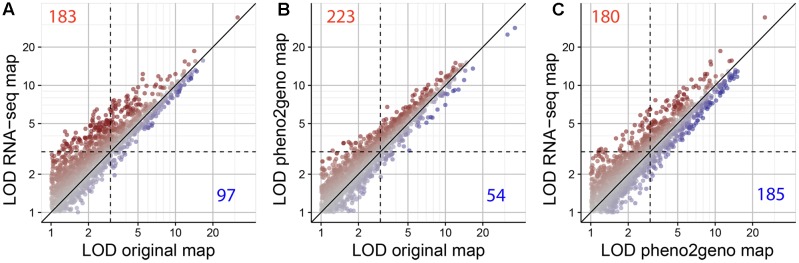
LOD score comparison of QTLs for 2550 QTL peaks of 510 published phenotypes using the original, the pheno2geno, and the RNA-seq map. **(A)** LOD scores with the RNA-seq map versus the original map, **(B)** LOD scores with the pheno2geno map versus the original map and **(C)** LOD scores with the RNA-seq map versus the pheno2geno map. The significance threshold is indicated by a dashed horizontal and vertical black line. “Stronger” LOD scores are plotted in red. Red and blue numbers correspond to the number of significant QTLs identified on the *x*-axis map with increased or decreased LOD scores in the *y*-axis map, respectively.

Quantitative trait locus mapping was performed to evaluate the mapping resolution of the RNA-seq map as compared to the two other maps. Using a genome-scan single QTL model analysis, 510 published phenotypes were re-mapped using the three maps. The QTL analysis with the RNA-seq map resulted in 754 significant QTLs (LOD > 3), while 684 and 568 significant QTLs were detected using the pheno2geno and the original map, respectively (**Table 3** and **Figure 7**). QTLs were considered to have a higher or lower LOD score if the difference between the compared LOD scores was larger than or equal to 0.5. Respectively, 223 and 183 of the total number of significant QTLs in the original map did show an increased LOD score in the pheno2geno map and RNA-seq map (**Figures 7A,B** and **Table 3**). When compared to the pheno2geno map, the RNA-seq map resulted in 180 QTLs with a higher LOD score (**Figure 7C** and **Table 3**). The pheno2geno map identified 139 new QTLs compared to the original map, while the RNA-seq map added 208 new QTLs. One hundred and twenty-five new QTLs were detected in the RNA-seq map as compared to the pheno2geno. In addition, an increase in the LOD scores was observed using the RNA-seq map as compared to the original map (average LOD score differences of 1.74) and the pheno2geno map (1.66) than for the pheno2geno compared to the original map (1.15) (**Table 4**). Together, these results indicate that the higher marker density of the RNA-seq map provides additional power to detect QTLs.

**Table 3:** Comparison of LOD scores using the different maps.

Genetic map^1^ (/compared to)	Significant QTLs (LOD > 3)	“New” and “lost” QTLs^2^	Higher LOD QTLs^3^	Lower LOD QTLs^4^
Original	568	–	–	–
pheno2geno/original	684	**139**/23 (**24%**/0.4%)	223 (39%)	54 (9.5%)
RNA-seq/original	754	**208**/22 (**30%**/0.4%)	183 (32%)	97 (17%)
RNA-seq/pheno2geno		**125**/55 (**18%**/8%)	180 (26%)	185 (27%)

^1^*The “new” maps used for the comparison are indicated in bold. ^2^New QTLs are the number of QTLs with a LOD score above 3 in the new map and below 3 in the compared map (bold numbers). These numbers are compared to the number of significant QTLs in the compared map “lost” in the new map. ^3^Higher LOD QTLs is the number of significant QTLs with a higher LOD score in the new map with a difference in LOD scores equal or larger than 0.5. ^4^Lower LOD QTLs is the number of significant QTLs with a higher LOD score in the new map with a difference in LOD scores equal or larger than 0.5. Percentage of new, lost, higher, and lower LOD QTLs in relation to the total number of significant QTLs in the compared map are shown in brackets*.

**Table 4:** Average LOD score differences across the different maps.

		A		
	Genetic maps	Original	Pheno2geno	RNA-seq
**B**	Original	–	1.15 (0.04)	1.74 (0.10)
	Pheno2geno	1.45 (0.19)	–	1.66 (0.12)
	RNA-seq	0.98 (0.04)	1.2 (0.04)	–

*Numbers indicate the average LOD score difference for QTLs with a higher LOD score using map A as compared to map B. Standard errors are indicated in brackets. The numbers of QTLs used for the analysis are reported in **Table 3** (see higher and lower LOD QTLs)*.

**FIGURE 8:**
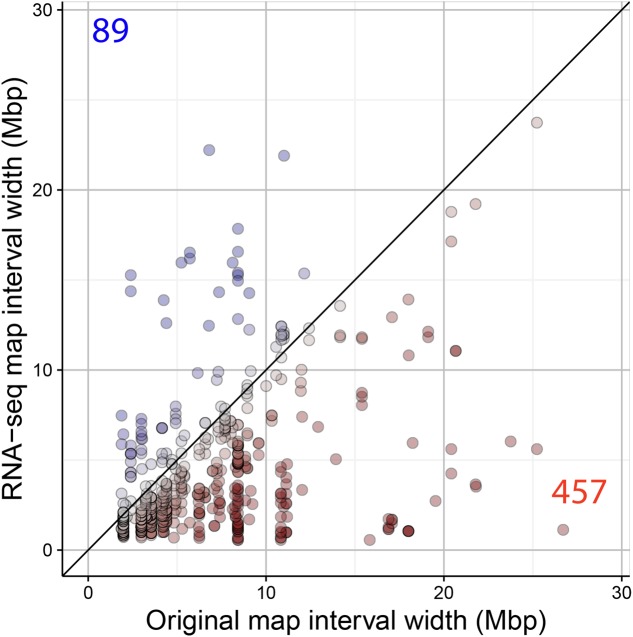
Comparison of the QTL mapping resolution using the original and the RNA-seq map. CIs (in Mbp) of QTLs detected in the original and the RNA-seq map are shown. Red and blue dots/values indicate the number of significant QTLs (LOD > 3) with reduced and increased CI in the RNA-seq map, respectively.

**FIGURE 9:**
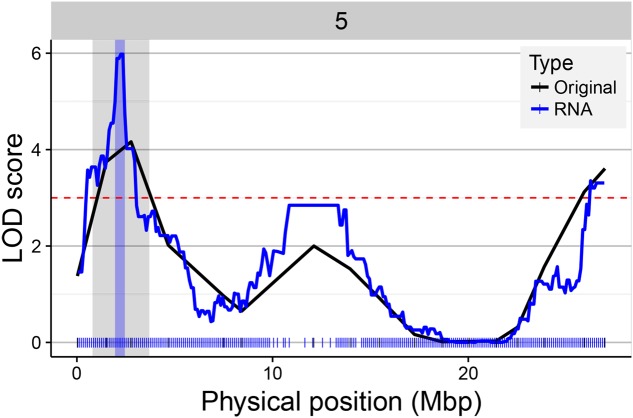
Gain in QTL mapping precision using the RNA-seq map. The figure illustrates the differences in LOD score and CI of the QTL for the trait “Size_circ_D_mei.10” located on the top of chromosome 5 using the original (black line) and the RNA-seq map (blue line). The physical position of the markers in the original and RNA-seq map is represented on the *x*-axis with black (17) and blue (243) tick marks, respectively. The QTL significance threshold is indicated by a horizontal dashed red line. The gray and blue vertical bars in the region of the QTL of interest indicate the CI of the QTL in the original and RNA-seq map, respectively.

A main factor for the success of QTL experiments is the precision in the estimation of the position of the QTL. We assessed the RNA-seq map resolution by comparing the CI of QTLs detected in the original map and the RNA-seq map. The CI of 546 QTLs significant in both maps was calculated (LOD > 3). Four hundred and fifty-seven (84%) of the QTLs showed a reduced interval in the RNA-seq map (**Figure 8**). The difference in interval width ranged from 0.08 to 25.58 Mbp. For example, the QTL for seed circularity at the top of chromosome 5 was delimited to a genomic region of less than 1.12 Mbp using the RNA-seq map compared to more than 26 Mbp using the original map (**Figure 9**). To verify the consistency of these results, the analysis was also conducted with a LOD threshold of 2 and for QTLs with higher LOD scores using the original map (Supplementary Figure S3). Eighty-one percent (770/952) of the QTLs showed a reduced CI using the RNA-seq map when the significance threshold was lowered to LOD > 2 (Supplementary Figure S3A). Analysis of 233 significant QTLs in both maps for which the LOD score was higher in the original map as compared to the RNA-seq map resulted in 72% (169/233) of these QTLs showing a reduced CI using the RNA-seq map (Supplementary Figure S3B). These results clearly show that the accuracy of the QTL mapping is improved by using the high-density SNP bin map.

## Discussion

### High-Density Genetic Map

In this study we showed that RNA-seq data can effectively be used for SNP calling, RIL genotyping, and the development of a high-density genetic linkage map. The used binning approach resulted in 1059 high-quality multi-SNP-based markers, providing a dense and equal coverage of markers physically anchored to the genome. The high marker density enabled more precise identification of recombination breakpoints and revealed unknown recombination breakpoints within the RIL population (**Table 2**). As a result, the mapping resolution is no longer limited by the number of markers but rather depends on the number of recombination events captured by the mapping population. This means that the advantages of high-density genetic maps in respect to mapping resolution will be considerably improved in combination with larger and/or more advanced designed populations (Balasubramanian et al., 2009; Kover et al., 2009; Liu et al., 2016). In comparison to the available genetic maps, the RNA-seq map could substantially increase QTLs linkage, eventually resulting in the identification of new QTLs (**Table 3**). Although the pheno2geno map showed a larger number of QTLs with higher LOD scores compared to the original map (**Table 3**), the RNA-seq map considerably increased the LOD scores of significant QTLs compared to both the original and the pheno2geno map (**Table 4**). Although we focussed in this study on the highest QTL per chromosome and per trait, we expect the RNA-seq map to also increase the overall number of QTLs after a more comprehensive analysis.

### Gain in QTL Mapping Resolution

The detection power and resolution of QTL mapping is significantly improved by high density genetic maps as compared to traditional markers (Yu et al., 2011). With the RNA-seq map, a major improvement was observed in the reduction of the LOD-1 CIs for 74% of the investigated QTLs. As a QTL CI in general encompasses a large number of genes, reduced CIs is of great benefit to narrow down the number of candidate genes for further investigation. In genetical genomics experiments, eQTLs can be identified as being either *cis*- or *trans*-regulated. Commonly, the distinction of both is made based on the distance, in cM or Mbp, between the gene and the eQTL peak or from the CI of the eQTL (Li et al., 2006, 2010; Keurentjes et al., 2007; West et al., 2007; Rockman et al., 2010; Terpstra et al., 2010; Vinuela et al., 2010; Aylor et al., 2011; Snoek et al., 2012, 2017; Lowry et al., 2013; Cubillos et al., 2014; King et al., 2014; Drost et al., 2015; Ranjan et al., 2016; Sterken et al., 2017). Therefore, gain in mapping precision is also likely to contribute to a more accurate identification of *cis*- versus *trans*-eQTLs.

### Advantages and Limitations of Using RNA-Seq Data

The use of RNA-seq presents several advantages over other methods. Our results show that RNA-seq data are a convenient and cost-effective source of SNP discovery, especially when a population is anyhow subjected to an eQTL analysis with the help of RNA-seq. RNA-seq can also overcome shortcomings identified from expression arrays based studies: while the effect of a SNP on the probe has enabled the identification of new sequence polymorphisms, weakened hybridization on microarrays based on expression studies can also cause the detection of false *cis*-eQTLs (Alberts et al., 2007; Chen et al., 2009). Furthermore, RNA-seq has the potential to study more complex levels of the genetic control of gene expression, for instance by quantification of alternative splicing (Filichkin et al., 2010; Yoo et al., 2016).

Single-nucleotide polymorphisms that are found with RNA-seq are inherently restricted to expressed exons, thus dependent on the developmental stage of the sequenced material and the experimental conditions. This restriction can also cause regions with low gene density or lowly expressed genes to be under represented. However, these disadvantages will often not affect the mapping due to the high number of intermediate to highly expressed genes in any tissue and the SNPs present in those genes. Although our approach finds variants that affect protein-coding sequences, it is largely blind to SNPs in promoters, introns, and intergenic regions. However, SNPs that are causal for phenotypic variation will often be found in or close to genes and therefore, SNPs in large non-genic regions will hardly result in improvements of quantitative traits mapping (Li et al., 2012). In view of the abundance and saturation of SNPs that were discovered in this study, this does not cause a disadvantage, but might limit SNP detection for crosses from nearly identical parents.

## Conclusion

This study demonstrates that RNA-seq data can effectively be used for SNP discovery and the development of high-density genetic linkage maps. Here we provide a new SNP-based saturated genetic map for a Bay × Sha RIL population. This saturated genetic map resulted in higher precision QTL mapping with more QTLs and considerably reducing the QTL CIs. Such improvements are of great benefit for the accurate mapping of more complex traits and the identification of causal genes.

## Author Contributions

WL designed the experiment. LW and ES grew the plants and provided the RNA samples. JJ-G processed the RNA samples and performed the RNA-seq analysis. HN processed the RNA-seq data and performed the SNP calling. LS developed and executed the binning and genotyping pipeline. ES analyzed the data and wrote the manuscript. HN, LS, JJ-G, HH, and WL read, revised, and agreed with the content of the manuscript.

## Conflict of Interest Statement

The authors declare that the research was conducted in the absence of any commercial or financial relationships that could be construed as a potential conflict of interest.

## Footnotes

1 http://www.ncbi.nlm.nih.gov/bioproject/418075

2 http://www.bioinformatics.nl/AraQTL/

3 http://publiclines.versailles.inra.fr/page/13
