# Supplementary material for: Construction of a High-Density Genetic Map from RNA-Seq Data for an Arabidopsis Bay-0 × Shahdara RIL Population
Source: Front Genet. 2017 Dec 5;8:201. doi: 10.3389/fgene.2017.00201 (PMC5723289; doi:10.3389/fgene.2017.00201)
Supplement: Supplementary file 1 [file Supplementary_Figures.DOCX]

**Construction of a High-density Genetic Map from RNA-seq Data for an Arabidopsis Bay-0 x Shahdara RIL Population**

**Serin E.A.R.^1#^, Snoek L.B.^2,3^****^#^****^*^, Nijveen H.^1,4^, Willems L.A.J.^1^, Jiménez-Gómez J.M.^5,6^, Hilhorst H.W.M.^1^, Ligterink W.^1*^**

^*^**Correspondence:**

Basten L. Snoek

[snoek.basten@gmail.c](mailto:snoek.basten@gmail.com)om

Wilco Ligterink

[wilco.ligterink@wur.nl](mailto:wilco.ligterink@wur.nl)

**Supplemental tables**

**Supplemental table S1:** Set of RILs used in this study

**Supplemental table S2:** Genetic map and genotyping data for the RNA-seq map using 160 RILs

**Supplemental table S3:** Cross object used for the QTL mapping with 1059 markers

**Supplemental table S4:** Output of the QTL mapping on 510 phenotypes for 145 RILs – LOD score comparison

**Supplemental table S5:** Output of the QTL mapping on 510 phenotypes for 145 RILs – Confidence interval comparison

**Supplemental figures:**

0

20

40

60

0

50

100

SNPs per 100 kbp bin

count

**Supplemental figure 1**: Distribution of the number of SNPs per 100 kbp bin.

**Supplemental figure 2:** Example of introgressions identified with the RNA-seq base map. The original (yellow) and SNP binned markers derived from the RNA-seq data were ordered based on their physical position. “A” in red and “B” in blue correspond to the Bay-0 and Sha genotypes, respectively. For the RIL 122 and the RIL 124, two new introgressions (black delineated regions) of 1 Mbp and 400 kbp respectively, were identified using the new markers. As a result, new recombination breaks are revealed (yellow crosses).


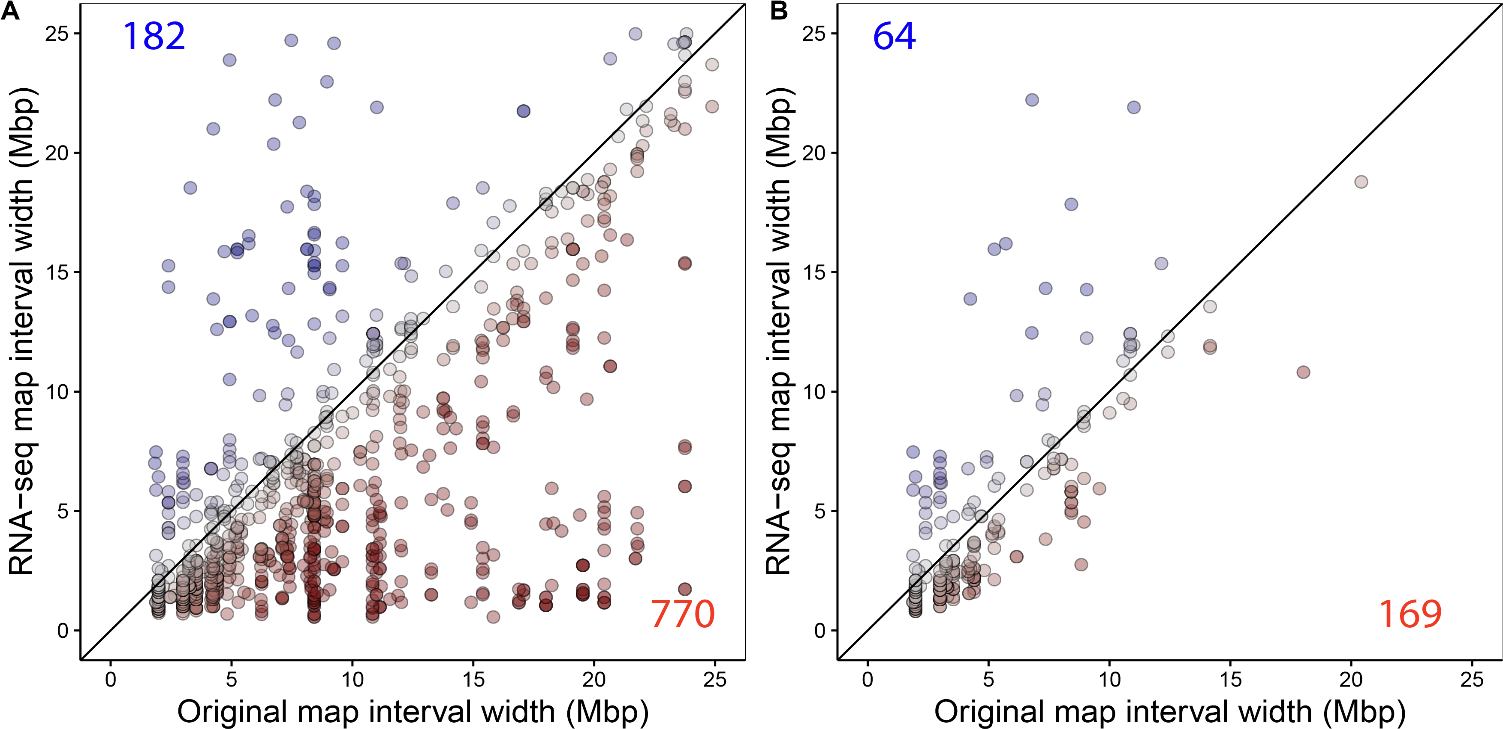


**Supplemental figure 3:** Comparison of QTL mapping resolution. Confidence interval width (in Mbp) of QTL peaks detected in the original and the RNA-seq map is shown. Red and blue dots indicate the number of QTLs with reduced and increased confidence interval in the RNA-seq map respectively of significant QTLs A) using a LOD significance threshold of 2 B) with a higher LOD score in the original map using a LOD significance threshold of 3.
